# Allelic Imbalance of Recurrently Mutated Genes in Acute Myeloid Leukaemia

**DOI:** 10.1038/s41598-019-48167-4

**Published:** 2019-08-13

**Authors:** Aarif M. N. Batcha, Stefanos A. Bamopoulos, Paul Kerbs, Ashwini Kumar, Vindi Jurinovic, Maja Rothenberg-Thurley, Bianka Ksienzyk, Julia Philippou-Massier, Stefan Krebs, Helmut Blum, Stephanie Schneider, Nikola Konstandin, Stefan K. Bohlander, Caroline Heckman, Mika Kontro, Wolfgang Hiddemann, Karsten Spiekermann, Jan Braess, Klaus H. Metzeler, Philipp A. Greif, Ulrich Mansmann, Tobias Herold

**Affiliations:** 1 Institute of Medical Data Processing, Biometrics and Epidemiology (IBE), Faculty of Medicine, LMU Munich, Munich, Germany; 2 Data Integration for Future Medicine (DiFuture, www.difuture.de), LMU Munich, Munich, Germany; 3 Laboratory for Leukemia Diagnostics, Department of Medicine III, University Hospital, LMU Munich, Munich, Germany; 4 Institute for Molecular Medicine Finland (FIMM), University of Helsinki, Helsinki, Finland; 5 Laboratory for Functional Genome Analysis (LAFUGA), Gene Center, University of Munich, Munich, Germany; 6 Institute of Human Genetics, University Hospital, LMU Munich, Munich, Germany; 7 Leukaemia and Blood Cancer Research Unit, Department of Molecular Medicine and Pathology, University of Auckland, Auckland, New Zealand; 8 Department of Haematology, Helsinki University Hospital Comprehensive Cancer Center, Helsinki, Finland; 9 German Cancer Consortium (DKTK), Partner Site Munich, Munich, Germany; 10 German Cancer Research Center (DKFZ), Heidelberg, Germany; 11 Department of Oncology and Hematology, Hospital Barmherzige Brüder, Regensburg, Germany; 12 Research Unit Apoptosis in Hematopoietic Stem Cells, Helmholtz Zentrum München, German Research Center for Environmental Health (HMGU), Munich, Germany

**Keywords:** Molecular medicine, Cancer genomics, Acute myeloid leukaemia

## Abstract

The patho-mechanism of somatic driver mutations in cancer usually involves transcription, but the proportion of mutations and wild-type alleles transcribed from DNA to RNA is largely unknown. We systematically compared the variant allele frequencies of recurrently mutated genes in DNA and RNA sequencing data of 246 acute myeloid leukaemia (AML) patients. We observed that 95% of all detected variants were transcribed while the rest were not detectable in RNA sequencing with a minimum read-depth cut-off (10x). Our analysis focusing on 11 genes harbouring recurring mutations demonstrated allelic imbalance (AI) in most patients. *GATA2*, *RUNX1*, *TET2*, *SRSF2*, *IDH2*, *PTPN11*, *WT1*, *NPM1* and *CEBPA* showed significant AIs. While the effect size was small in general, *GATA2* exhibited the largest allelic imbalance. By pooling heterogeneous data from three independent AML cohorts with paired DNA and RNA sequencing (N = 253), we could validate the preferential transcription of *GATA2*-mutated alleles. Differential expression analysis of the genes with significant AI showed no significant differential gene and isoform expression for the mutated genes, between mutated and wild-type patients. In conclusion, our analyses identified AI in nine out of eleven recurrently mutated genes. AI might be a common phenomenon in AML which potentially contributes to leukaemogenesis.

## Introduction

Genomic alterations in cancer are heterogeneous and complex but mainly thought to disturb protein function or gene expression^1^. The extent to which such mutations are transcribed into RNA is largely unknown. One of the main reasons for this lack of knowledge is due to the intrinsic complexity of transcriptome sequence data, which makes it difficult to implement variant calling procedures^2^. Thus, variant identification from RNA sequences (RNA-Seq) is considered inferior to that from DNA sequences (DNA-Seq). Recent developments in computational algorithms address these issues and established splice-aware alignment of transcriptome sequences in an effective manner^3,4^. The choice of the aligner and variant caller has a major influence on variant detection^5–7^. In addition, finding insertions and deletions (INDELs) in RNA-Seq is still one of the major challenges due to the complexity of RNA splicing^8^. The RNA variants can be compared with the variants from DNA to determine the reliability of RNA-Seq analysis pipelines for variant discovery^8,9^.

O’Brien and colleagues compared whole exome sequencing (WES) and RNA-Seq data from 27 lung cancer pairs of tumour and matched normal samples and found only 14% overlap among single nucleotide variants (SNVs) detected^10^. In contrast, another group observed 99% concordance of somatic mutations detected between DNA- and RNA-Seq in an analysis of mouse tumour cell lines^11^. They also examined the allelic imbalance (AI) and concluded that mutated and wildtype alleles were expressed equally irrespective of their mutation status. Few other studies have looked at the AI of somatic mutations between DNA and RNA^12,13^. Rhee and colleagues analysed the AI of somatic mutations in the cancer genome atlas (TCGA) cohort from five human solid tumour types and found differences in allele-specific expression among splice site mutations, nonsense SNVs and frameshift INDELs^12^. TCGA reported allelic biases in the expression of mutations in *DNMT3A*, *RUNX1*, *TET2*, *TP53*, *WT1* and *PHF6* between paired DNA- and RNA-Seq data in acute myeloid leukaemia (AML) samples^13^. Despite the limitation of this analysis due to low mutation counts in the cohort, AI could be explained by copy number changes, loss of heterozygosity or hemizygosity in the case of *PHF6*. However, the higher expression of the mutant alleles could not be explained sufficiently in all other cases^13^. Celton *et al*. studied the expression levels of *GATA2* among normal karyotype AML samples and observed the existence of allele-specific expression in samples with low *GATA2* expression and further demonstrated an increased DNA methylation in the lower expressed allele^14^.

Although the phenomenon of AI was observed in different cancer types, there is no systematic analysis or validation of such imbalances for recurrently mutated genes in AML. In our study, we examined the correlation between DNA and RNA Variant Allele Frequencies (VAFs) of recurrently mutated genes in 499 AML patients to determine AI. In contrast to previous analyses, we were able to compare high coverage DNA and transcriptome sequences in a large and homogenously sampled patient cohort and validate our findings in independent data sets. We identified a subgroup of genes that showed AI which potentially contributes to the pathogenic effect of these mutations.

## Results

**Figure 1:**
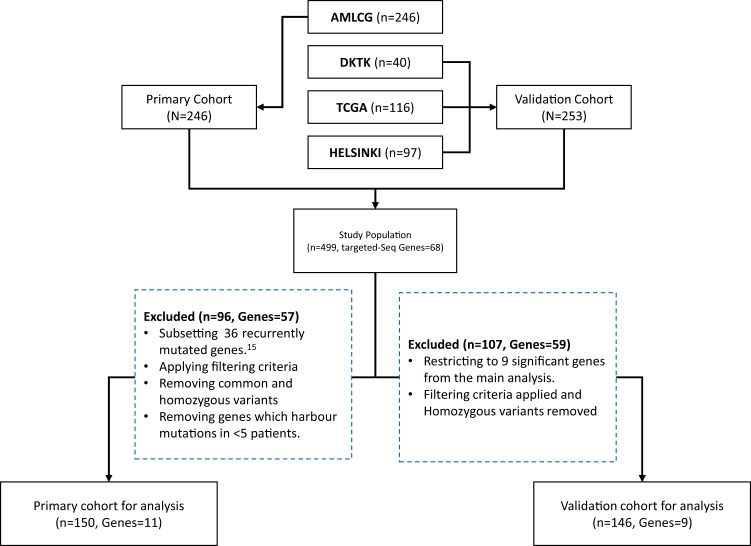
Flow diagram of primary and validation cohorts. The dotted blue boxes indicate general criteria applied on excluding genes and samples. The 11 genes included in the analyses were *PTPN11*, *U2AF1*, *IDH2*, *FLT3*, *SRSF2*, *TET2*, *RUNX1*, *GATA2*, *CEBPA*, *WT1* and *NPM1*, respectively.

Our analysis included 499 adult AML patients from four independent cohorts with paired DNA- and RNA-Seq data. The AMLCG cohort (N = 246) was used as the discovery cohort. We focused on 36 genes which were recurrently mutated in more than 1% of AML patients^15^. Out of those, only 11 genes met our filtering criteria and were examined further (Fig. 1). The alignment and variant calling pipeline is shown in Supplementary Fig. S1. The effect of adapter trimming and quality filtering of DNA- and RNA-Seq in the AMLCG cohort is shown in Supplementary Figs. S2 and S3, respectively. The mean coverage of the regions of interest in the AMLCG data set among the targeted DNA- and RNA-Seq were 542x and 85x, respectively. Detailed alignment information are listed in Supplementary Table S1. Further, variant calling procedures were applied to extract putative somatic mutations which were used for downstream analyses.

### Raw variants and read depth

**Figure 2:**
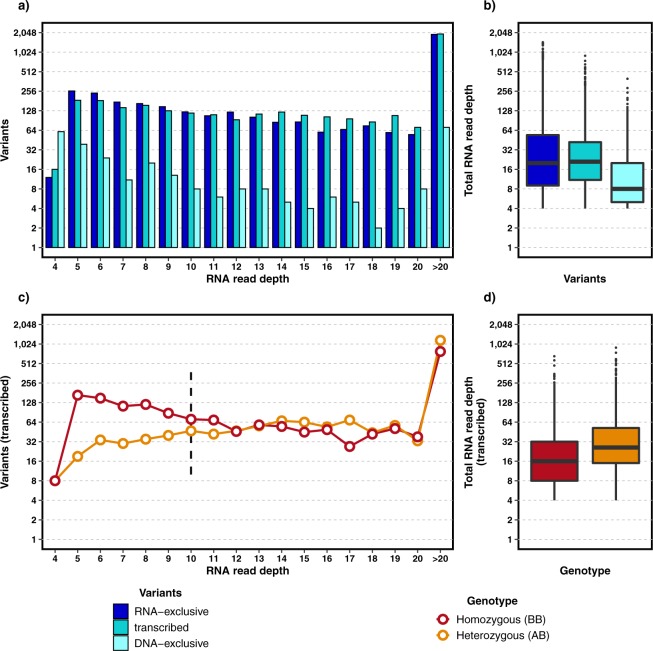
RNA-Seq read depths of all detected variants. (**a**) RNA-Seq read depths grouped based on the different variant classes. (**c**) RNA-Seq read depth of transcribed variants (variants detected in both DNA and RNA) grouped according to variant genotype information. (**b**,**d**) Read depth distribution based on variant groups.

We set out to determine the allele-specific transcript abundance by calling variants and classifying them into three groups: transcribed (present in both DNA and RNA), DNA-exclusive variants (not detected in RNA with minimum read depth of 4x) and RNA-exclusive variants (not detected in DNA with minimum read depth cut-off of 30x). The variants were called in the recurrently mutated regions in AML as defined in previous studies (Supplementary Table S2)^15,16^. The RNA-Seq variants were binned based on their read depth (Fig. 2). There were 8,052 variants called in the defined regions from both sequences including DNA-exclusive and RNA-exclusive variants (variants in both sequences were counted once, 89.3% were SNVs and 10.7% were INDELs). A large number of variants were RNA-exclusive (47.9%) most of which are likely to be false positives due to sequencing errors (Fig. 2a,b), while a minority may be the result of RNA editing. On the other hand, only a small number of variants were DNA-exclusive (3.8%). In Fig. 2a and Supplementary Fig. S4, the number of DNA-exclusive variants decreases with the increase in the RNA read depth. However, only a modest decrease could be observed in the case of RNA-exclusive variants. On the other hand, the proportion of transcribed variants also tend to vary across different RNA read depths, necessitating an appropriate minimum read depth cut-off in RNA-Seq. To select a suitable cut-off, we calculated the proportions between homozygous (BB) and heterozygous (AB) genotypes for all transcribed variants (Fig. 2c,d). With increasing RNA read depth, we observed a convergence of the homozygous and heterozygous proportions and the difference between them stabilized above a read depth of 10. Interestingly, TCGA also considered a minimum read depth of 10x to detect variants in RNA-Seq^13^. However, the number of RNA-exclusive variants did not show a considerable drop-off even in regions with high coverage in RNA-Seq (Fig. 2a). SNVs and INDELs showed noticeable differences, mainly due to the differences in their variant counts per read depth. Also, large number of somatic INDELs were heterozygous, which makes it difficult to assume similar proportions of homozygous and heterozygous variants (Supplementary Fig. S4).

### Filtering variants

Using a minimum read depth cut-off on called variants did not sufficiently remove the large number of potential false positive variants caused by sequencing biases due to mapping quality, base quality, variant position in the aligned reads etc. (Supplementary Fig. S5). Applying additional filtering criteria accounting for these biases, error-prone regions, RNA editing sites and repeat regions excluded 36.2% of all transcribed variants, leaving 2302 SNVs and 182 INDELs (Supplementary Fig. S6). The reduction was more prominent among DNA- and RNA-exclusive variants (59.7% and 99.4%, respectively). Almost all potential false positives were removed in the case of RNA-exclusive variants.

### DNA and RNA variant comparison

**Figure 3:**
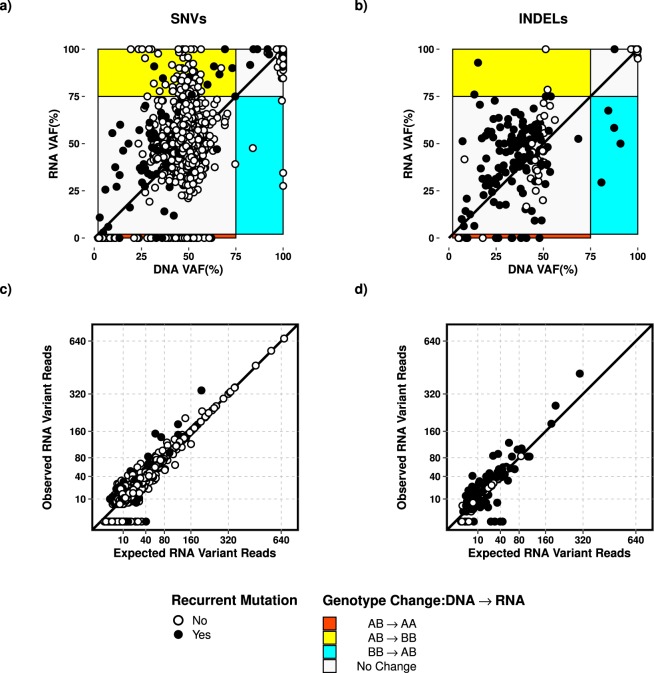
Variant allele frequency differences of transcribed and DNA-exclusive variants (2,606) including recurrent mutations (284) for SNVs (**a**) and INDELs (**b**). Expected and observed RNA variant read depths of SNVs (**c**) and INDELs (**d**). The diagonal lines represent the expected DNA vs. RNA trend in terms of VAFs (**a**,**b**) and RNA variant read depths (**c**,**d**). The genotype conversion of AB → AA and AB → BB represent the allele specific transcript abundance of wild-type and mutant allele, respectively. The observation of BB → AB genotype change artefacts might be due to the arbitrary definition of homozygous and heterozygous variants. We excluded regions with DNA VAF < 2% and regions with BB → AA genotype change.

After minimizing the number of potentially false positive variants, we set out to determine the variability of VAF among transcribed (2,484) and DNA-exclusive (122) variants, in the remaining 2,606 variants. Of the variants detected in DNA-Seq, 95.4% were also found in RNA-Seq (transcribed variants). Our observations based on genotype information alone showed that 92.3% of all filtered variants display no observable changes in VAFs between DNA and RNA sequences (Fig. 3a,b). The observed VAFs of recurrent mutations in genes commonly affected in AML also showed a similar trend (83.5%). About 5.3% of mutated alleles were over-represented in RNA-Seq (variants with heterozygous mutant alleles in DNA and homozygous mutant allele in RNA) while we were unable to detect 9.9% of the recurrent mutations (at 10x coverage), which were detected in the DNA-Seq data, indicating a lack of transcription.

**Figure 4:**
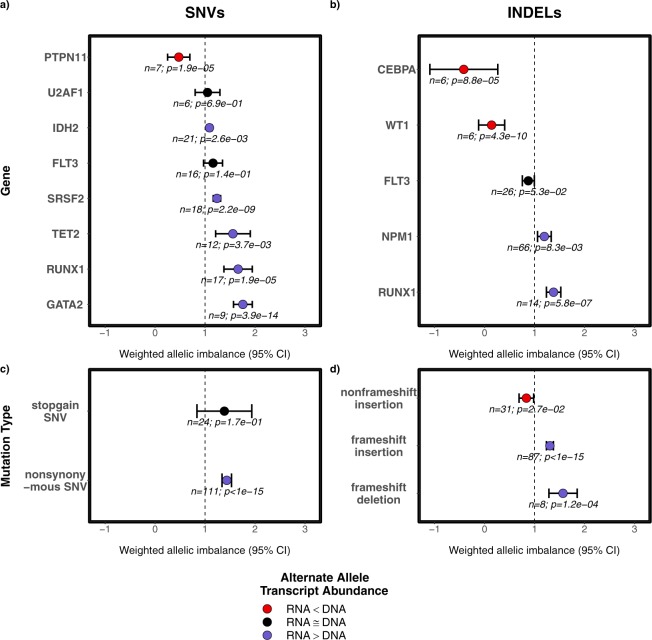
Weighted allelic imbalance (WAI) of recurrent mutations per gene in the AMLCG cohort for SNVs (**a**) and INDELs (**b**). WAI of recurrent mutations per mutation type in the AMLCG cohort for SNVs (**c**) and INDELs (**d**). The dotted vertical line at WAI of 1 indicates no allelic imbalance among the variants in DNA and RNA. WAI ≥ 1 indicates preferential mutant transcript abundance and WAI ≤ 1 represents preferential wild-type transcript abundance.

All heterozygous mutations in genes with at least 5 heterozygous mutations in their exonic regions were extracted and included in a regression model (see methods) for SNVs and INDELs to determine the weighted allelic imbalance (WAI). The model on SNVs showed a substantial imbalance towards wild-type transcript abundance for *PTPN11*, whereas considerable imbalances towards mutant transcript abundance was observed for *GATA2*, *RUNX1*, *TET2*, *SRSF2* and *IDH2* (Fig. 4). On the other hand, INDELs in *CEBPA* and *WT1* showed a noticeable WAI towards wild-type allele. Also, we detected the opposite effect in the case of *NPM1* and *RUNX1* INDELs in which the WAI tend towards increased mutant allelic abundance in RNA. The VAF of mutations in *U2AF1* and *FLT3* (both ITD and TKD mutations) remained stable between DNA and RNA in all patients. The effect of mutation type on the AI was also observed (Fig. 4c,d). Non-synonymous SNVs and frameshift INDELs showed a higher imbalance towards the mutant transcript abundance while non-frameshift insertions showed a trend towards the wild-type allele abundance in RNA. Surprisingly, stop/gain SNVs showed no signs of AI among the mutations analysed.

### Weighted allelic imbalance in external validation cohorts

*GATA2* mutations showed the highest allele-specific mutant transcript abundance in our cohort, but *GATA2* mutations are rare in AML. To validate our results, we pooled *GATA2* mutated samples along with samples harbouring mutations in 8 other genes of interest from external data sets with paired DNA- and RNA-Seq data (DKTK, TCGA and HELSINKI). The WAI analysis was modified to account for the differences in cohorts (methods).

**Figure 5:**
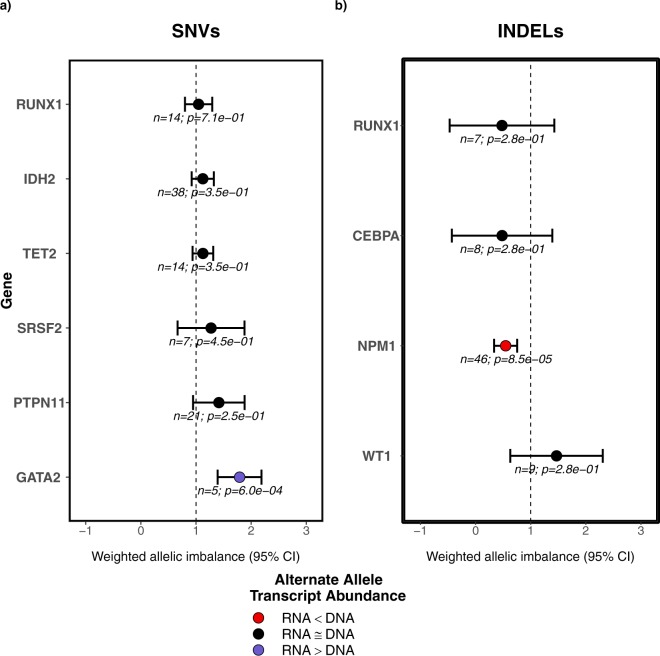
Weighted allelic imbalance of recurrent mutations per gene among the pooled DKTK, TCGA and HELSINKI cohorts for SNVs (**a**) and INDELs (**b**).

We were able to validate the significant shift of AI towards mutant allelic abundance in RNA for *GATA2*, suggesting consistent preferential transcript abundance (Fig. 5). Different from what we had observed in our discovery cohort, *NPM1* showed an allelic imbalance towards wild-type abundance. It is to be notated that *NPM1* had a very low effect size (i.e. very small AI) in the discovery cohort. The rest of the genes showed no significant AI.

### Weighted allelic imbalance based on SNP analysis

**Figure 6:**
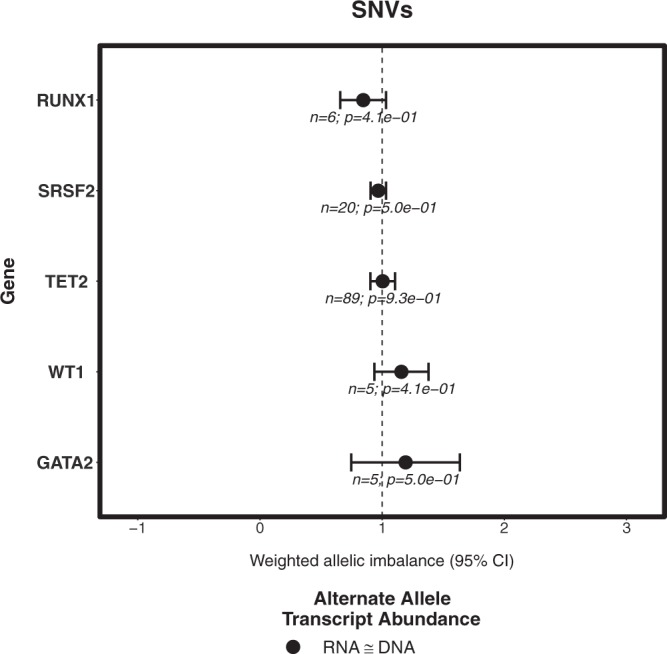
Weighted allelic imbalance of common SNPs in the AMLCG cohort without recurrent mutations in the respective genes.

We extended our investigation to patients without recurrent mutations in the genes of interest (nine genes which showed significant AI in our main analysis), to determine if they also show allele-specific transcript abundance in AML. All common SNPs from the AMLCG cohort were extracted and filtered using the criteria we previously established (Supplementary). We then extracted all dbSNP annotated variants (build 138, NonFlagged) and performed our WAI analysis to compare the minor allele frequencies (MAFs) of the common variants (Supplementary Table S3). The analysis was restricted to five genes with significant AI and at least 5 SNPs in the pooled data set. We did not find any AIs for SNPs among the selected genes (Fig. 6).

### Internal validation of allele-specific transcript abundance

**Figure 7:**
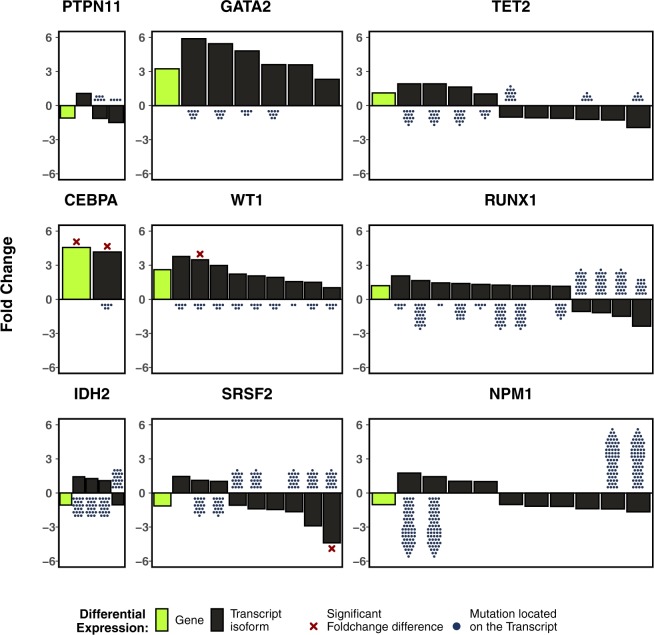
Gene-level and transcript-level differential expression calculated with limma after precision-weighting with voom for all recurrently mutated genes with a significant WAI in the AMLCG cohort. The green boxes indicate gene fold change and black boxes indicate different transcript isoforms. Dots below or above the bars represent recurrent mutations present within the transcripts. Crosses represent significant fold change differences (adjusted p value < 0.05). This plot is to provide a visual representation of significant fold change difference and the location of mutations within the transcripts and thus the transcript identifiers were removed.

Except for *CEBPA*, no other gene with significant WAI showed noticeable differential transcript abundance in our primary cohort between patients harbouring recurrent mutations in that gene and patients without mutations in the gene. Differential expression of transcript isoforms revealed one isoform in each of *CEBPA*, *WT1* and *SRSF2*, to be differentially expressed based on the mutation status of those genes. However, the presence of these mutations was not restricted to these transcript isoforms alone. Other transcript isoforms in *WT1* and *SRSF2*, also harbouring the recurrent mutations, did not show any substantial differential expression between mutated and wild-type patients. It is therefore highly unlikely that the differential isoform expressions observed in *WT1* and *SRSF2* can be explained by mutations in the respective genes (Fig. 7). In the case of *CEBPA*, there was only one transcript with sufficient read counts to be considered for the analysis.

## Discussion

Only few studies have systematically investigated the difference of allele specific transcript abundance of genes with recurrent mutations in matched DNA and RNA sequencing samples so far^11–13^. We analysed a large cohort of AML patients with DNA and RNA sequence information and identified allele specific transcript abundance in 9/11 recurrently mutated genes.

One of the major advantages of quantifying imbalances of transcript abundance in a uniform cohort lies in the reduction of ascertainment bias, which in turn improves the validity of the results. Studies comparing WES to transcriptome sequencing defined AI using the allelic fraction difference (RNA VAF minus DNA VAF)^11,12,17,18^. This method is not appropriate when comparing targeted DNA-Seq to RNA-Seq (as in our case) due to the vast differences in sequence coverage. We addressed this issue by transforming the VAFs of both sequences into expected and observed mutant allele reads ensuring their comparability.

A major drawback of using RNA-Seq for variant calling is the inherent low coverage in regions of interest, when compared to targeted DNA-Seq. Nevertheless, in accordance with previous publications we show that it is possible to validate the majority of the genomic variants using RNA-Seq (95.4%)^9^. However, RNA-Seq still remains unsuitable for variant discovery due to the large number of false positive variant calls (>52% in our analysis). To control for this, it is essential to select an ideal read depth cut-off. We approached this issue by optimizing the parameters for variant calling in DNA-Seq and using less stringent parameters for RNA-Seq to avoid the loss of true positive variants. We then visualized the concordance rate of homozygous and heterozygous variants with respect to incremental RNA read depths assuming similar proportions of the called variants. Indeed, the proportions of SNVs converged at 10x and remained stable at higher read depths, showing that 10x read depth is a reliable cut-off for RNA-Seq SNV calling, which is in agreement with the cut-off defined by Ley *et al*. for TCGA^13^. Similarly, Quinn *et al*. showed 89% specificity in calling SNPs at 10x cut-off^19^. Although a cut-off of 10x for RNA-Seq seems to be sufficient for variant calling, there is a potential bias to be addressed in the case of gene mutations which are often sub-clonal. As an example in the case of *PTPN11* mutations, VAF in DNA-Seq is usually low (median «50%) and thus a 10x read depth cut-off might not be ideal to confidently call the mutations or observe lack of transcription in RNA-Seq^15^. In contrast to SNVs, we were not able to define a reliable cut-off for INDELs due to their lower distribution per read depth in RNA-seq.

We observed preferential transcript abundance in nine genes (Fig. 4) that were found to be recurrently mutated in AML. Interestingly, six of them showed a significant (p < 0.05) increase in weighted AI towards the mutant allele, with *GATA2* exhibiting the largest difference. Such preferential mutant allele transcript abundance has been observed before in low *GATA2* expressing specimens of normal karyotype AML^14^. In the same study, the involvement of epigenetic mechanisms in allele-specific transcript abundance was demonstrated as well^14^. A similar observation of mono-allelic expression of the mutant allele of *GATA2* was made by Al Seraihi *et al*.^20^. The down-regulation of *GATA2* expression was shown to be a decisive step in the progression of leukaemia by transcriptional analysis in mouse models^21^. Ley *et al*. showed preferential allelic transcript abundance of *RUNX1* and *TET2* and preferential transcript abundance of the wild-type allele of *WT1* in an analysis of the TCGA cohort, which is consistent with our analysis^13^.

Some of the results can potentially be explained by the difference in the half-life of the RNA transcript of mutated and wild-type alleles, resulting from differential transcript stability. The phenomenon of uniparental disomy, copy number alterations or genomic imprinting might also be responsible for some AIs. Regardless of the mechanism, our results show small but significant imbalance in the transcription towards certain alleles. This does not seem to be random since it only occurs in genes affected by recurrent alterations. The WAI analysis based on SNPs showed no AI, in AML patients who did not harbour recurrent mutations in the genes. This observation implies an association of the presence of mutations and AI in these recurrently mutated genes.

Irrespective of the sequencing techniques and cohorts we studied, we were able to independently validate the effect for *GATA2* mutations in our pooled validation cohort mainly due to their larger effect size when compared to other genes. The differences in the preferential allelic transcript abundance of mutant versus wild-type alleles among the primary and validation cohorts in *NPM1* might be due to its smaller effect size in the primary cohort. Minor technical differences such as library preparations might have also prevented us to validate such small effect sizes in *NPM1* and other genes.

While we were able to show an effect of recurrent mutations on allele-specific transcript abundance in AML, we did not detect differential expression of transcript isoforms between mutated and non-mutated patients. Specifically, the recurrent mutations observed in the differentially expressed transcripts isoforms were also present in transcripts that showed no relevant differential expression between the two groups. This observation is not compatible with the simplistic assumption that differential expression seen in patients with mutated and non-mutated genes can be solely attributed to its mutation status. Thus, it remains unclear which additional factors contribute to the observed AI. Nevertheless, a differential isoform transcript expression in mutated and non-mutated patients can be detected in three genes harbouring recurrent mutations and may imply a reduced expression of mutant alleles or may be the effect of counteracting mechanisms in the case of preferential wild type allelic abundance observed in *SRSF2*.

Our analysis on mutation types showed that frameshift INDELs have an increased mutant allele abundance which contradicts Rhee *et al*.’s analysis, that demonstrated a tendency for negative allelic fraction differences^12^. Furthermore, we were unable to validate the negative allelic fraction difference among stop-gain SNVs observed by Rhee *et al*.^12^. Our results regarding stop-gain SNVs was also not compatible with known biological mechanisms such as nonsense-mediated RNA decay^22^. This might be due to the differences in sequencing techniques used in the studies. Rhee *et al*. compared RNA-Seq with WES, whereas we used targeted DNA-Seq^12^. Another explanation could lie in the different tumour types analysed and completely different genes included in each study. Rhee *et al*. included five different tumour types (Breast invasive carcinoma [BRCA], Head and Neck squamous cell carcinoma [HNSC], Kidney renal clear cell carcinoma [KIRC], Lung adenocarcinoma [LUAD] and Stomach adenocarcinoma [STAD] from TCGA) to determine the AI among the somatic mutations (AML was not included)^12^, thus suggesting a varying allele-specific expression between different genes and tumour entities. We tried to address this by including gene and mutation type interactions in our regression model but were unable to proceed further due to the few numbers of mutations.

The impact of AI on oncogenesis is unclear and may vary between different variants and diseases but it is tempting to speculate that the changes in expression enhance the impact of the underlying gene alteration (e.g. increasing the effect of a gain of function mutation). At the moment, sufficient data is missing to determine the incidence of this phenomenon. Functional analyses are a technically demanding challenge that can only be partially addressed by current routinely applied molecular methods. Potentially, more sophisticated tools to regulate gene expression output levels in mammalian cells will be able to address this question in the future^23^.

In summary, we demonstrated the existence of allele-specific transcript abundance among some of the recurrently mutated genes under study in AML. We suggest that the preferential transcription of wild-type or mutant alleles could be a common and under-appreciated phenomenon in AML and further research will be required to determine the potential effect of allele-specific transcript abundance in AML pathogenesis.

## Methods

### Study population

Our primary cohort consist of German AML Co-operative Group (AMLCG) study participants, sampled at initial diagnosis from 1999 and 2008 trials (n = 246). Details regarding the treatment protocols and patient selection were published previously^15,16^. Our validation cohorts include patients from DKTK (n = 40), TCGA (n = 116) and HELSINKI (n = 97)^13,24–26^. We only included patients having both DNA and matched RNA sequencing as well. Also, we restricted to 36 genes which were recurrently mutated in more than 1% of the AML patients by Metzeler *et al*.^15^. A summarized flow diagram with inclusion and exclusion of samples is shown in Fig. 1.

### DNA and RNA sequencing

A total of 246 samples (AMLCG) underwent DNA sequencing using a custom amplicon-based targeted enrichment assay (Haloplex, Agilent, Boeblingen, Germany) of 68 genes, which are recurrently mutated in AML^15,16^. The samples were sequenced paired-end (2 × 250 bp) on an IlluminaMiSeq instrument (Illumina, SanDiego, CA). Additionally, Whole Transcriptome Sequencing (Lexogen SENSE mRNA-Seq kit V2) was performed using a paired-end (2 × 100 bp), strand-specific, poly(A)-selected protocol^16^. Downstream analyses of both sequencing procedures included adapter clipping and quality trimming and was followed by sequence alignment. DNA and RNA sequences were mapped to the reference genome (hg19), using the BWA-MEM and the STAR aligner respectively^3,27^. In the case of RNA-Seq, sequence duplicates were removed after the alignment procedure. After processing the aligned sequences, SNVs were called using VarScan2, while INDELs were called using VarDict^28,29^. A detailed descriptions of both pipelines can be found in the supplementary methods and in Supplementary Fig. S1. In the case of the pooled validation cohorts (DKTK, TCGA and HELSINKI), details of WES are described in previous publications^13,24,25^. The sequence variants from both DNA and RNA were called using a variant calling pipeline similar to the one used in our primary cohort. Since raw sequencing files (fastq) were not accessible for TCGA and HELSINKI cohorts, the alignment files (bam) were integrated directly into the variant calling. The main difference in the variant calling procedure between the primary and the validation cohorts was the minimum read depth cut-off for DNA-Seq (10x when compared with our primary cohort 30x). This difference is due the differences in the sequencing methods.

### Criteria for variant filtering

Several filtering criteria, including read depth, strand bias, mapping and base quality biases, position bias etc. along with custom filtering definitions (Supplementary), were applied to find the optimum balance between eliminating false positives variants and retaining true positives (Supplementary Fig. S5). In our RNA-Seq pipeline, we lowered the threshold of the variant callers’ filtering parameters to avoid the elimination of putative variants.

### Statistical analysis

Recurrent mutations identified in our previous analysis, were selected from our dataset and their VAFs were compared between DNA- and RNA-Seq^15,16^. The genotype status of the alterations with allele frequencies between 2% and 75% were defined as heterozygous mutations. All homozygous mutations as well as RNA-exclusive variants were not included in the analysis. Linear regression models (1–3) including the observed and the expected RNA variant read depth in sequence fragments were used to determine the weighted allelic imbalance (WAI) of the mutations. We used a bootstrap approach (which does not rely on Gaussian distribution) to infer the statistic p-value and confidence intervals^30^. According to these models, an estimation of significant difference (p-value < 0.05) between the observed and expected RNA variant depth among variants in a gene indicate the presence of WAI in the respective gene. We defined the expected RNA variant read depth as follows:

$${RNA}\,{Variant}\,{Dept}{{h}}_{i{,}\mathrm{Exp}}={DNA}\,{VA}{{F}}_{i{,}\mathrm{Obs}}\ast {RNA}\,{Total}\,{Dept}{{h}}_{i{,}\mathrm{Obs}}/{\rm{100}}$$

And the following linear regression models

For every gene in the primary cohort:

1 $${RNA}\,{Variant}\,{Dept}{{h}}_{i{,}{Obs}} \sim {RNA}\,{Variant}\,{Dept}{{h}}_{i{,}\mathrm{Exp}}+{Mutation}\,{Typ}{{e}}_{{i}}$$

For every mutation type in the primary cohort:

2 $${RNA}\,{Variant}\,{Dept}{{h}}_{i{,}{Obs}} \sim {RNA}\,{Variant}\,{Dept}{{h}}_{i{,}\mathrm{Exp}}+{Gen}{{e}}_{{i}}$$

For every gene in the validation cohort:

3 $${RNA}\,{Variant}\,{Dept}{{h}}_{i{,}{Obs}} \sim {RNA}\,{Variant}\,{Dept}{{h}}_{i{,}\mathrm{Exp}}+{Mutation}\,{Typ}{{e}}_{{i}}+{Cohor}{{t}}_{{i}}$$

Model (1) grouped the mutations for each gene separately and was adjusted for mutation types such as synonymous and non-synonymous SNVs, stop/gain SNVs, frameshift and non-frameshift insertions, deletions and substitutions, in order to determine the possible effect of mutations on the difference of VAF between DNA and RNA. Each mutation pair (DNA and RNA) was considered as individual entity in the regression model even in the case of patients with multiple mutations on the same gene. Model (2) grouped the mutations by mutation type and was adjusted for gene as well. The regression models were applied on SNVs and INDELs separately. We also applied Model (1) on common SNPs in patients without recurrent mutations on the respective genes to determine the existence of allele-specific expression in general, irrespective of the mutational status. Model (3), modified from Model (1), was used to calculate the WAI in the validation cohort.

### Differential expression of genes and transcripts

The AI and allele-specific expression of recurrent mutations were further investigated by differential expression of genes and transcripts. The transcript quantification and aggregated gene quantification of our cohort was carried out using Salmon (v0.9.1)^31^. The quantified read counts with less than one count per million in five samples were filtered out and the rest were normalized (TMM) using edgeR (v3.20.9)^32^. They were then grouped based on the recurrent mutations of each gene with substantial WAI and the differential expression of those genes and transcripts were analysed using limma (v3.34.1) with sample-specific quality weight adjustments in the experiment design (voomWithQualityWeights)^33^. The fold changes were calculated and were adjusted for multiple testing.

All the processing of DNA- and RNA-Seq were carried out on an in-house Galaxy platform (v15.10.2)^34^. All statistical analyses were performed using R (v3.4.3) and were adjusted for multiple testing using Benjamini & Hochberg procedure^35,36^. We considered an adjusted p-value cut-off of 0.05 as significant.

### Ethical approval and informed consent

Study protocols were approved by the institutional review boards of the participating centers. All study protocols were in accordance with the Declaration of Helsinki, the ethical standards of the responsible committee on human experimentation (written approval by Ethikkommission bei der LMU München, number 427-13) and were approved by the institutional review boards of the participating centers. All patients provided written informed consent for inclusion on the clinical trial and in the genetic analyses.

## Supplementary information

Supplementary: Allelic Imbalance of Recurrently Mutated Genes in Acute Myeloid Leukaemia

## Data Availability

The gene expression data are publicly available through the Gene Expression Omnibus Web site (GSE106291). Due to law restrictions the sequence information cannot be made publically available but controlled access can be provided upon request.
