# Supplementary material for: Allelic Imbalance of Recurrently Mutated Genes in Acute Myeloid Leukaemia
Source: Sci Rep. 2019 Aug 13;9:11796. doi: 10.1038/s41598-019-48167-4 (PMC6692371; doi:10.1038/s41598-019-48167-4)
Supplement: Supplementary file 1 — Supplementary: Allelic Imbalance of Recurrently Mutated Genes in Acute Myeloid Leukaemia [file 41598_2019_48167_MOESM1_ESM.docx]

**Allelic Imbalance of Recurrently Mutated Genes in Acute Myeloid Leukaemia**

Batcha et al.

# Supplementary Methods

## Sequence Processing and Variant Calling

### Targeted DNA Sequencing

The Illumina adapters from the paired-end DNA sequences (length 250bp) were removed and the sequences were quality trimmed using Trimmomatic (v0.32).^1^ Trimmed reads were aligned to the reference genome build (hg19) using BWA-MEM algorithm.^2^ The INDELs were realigned using GATK toolbox (v2.7.4), followed by Single Nucleotide Variant (SNV) and Insertion and Deletion (INDEL) calling using VarScan (v2.3.5) and VarDict, respectively.^3–5^ We used VarDict for calling INDELs due to its better performance in calling INDELs in both DNA- and RNA-Seq (Supplementary Fig. S7). This comparison is made only with the default parameters of both tools. In the case of our analysis, a VarScan p-value of 0.01 was considered as a cut-off for preliminary filtering. Minimum read depth of 30x, mapping quality of 10, base quality of 20 and minor allele frequency >2% were considered for both variant callers. Functional annotation of these sequence variants were carried out using Annovar.^6^ Public databases, such as COSMIC, dbSNP, ClinVar and 1000 genomes project were used for annotation.^7–10^

### Transcriptome Sequencing

In case of paired-end RNA Sequencing (length 100bp), 9 and 6 bases were removed from the forward and reverse reads, respectively, in order to reduce the proportion of errors due to non-specific hybridization as recommended by the manufacturer.^11^ The adapters were then removed using Cutadapt (v1.7.1), followed by quality trimming.^1,12^ Sequences were then aligned to the reference genome (hg19) using splice-aware STAR aligner (v2.5.1b).^13^ Initial alignment was performed to collect the splice junctions from the cohort. These splice junctions from aligned samples were then pooled and the mitochondrial regions were filtered out. All known splice junctions were also filtered out in order to avoid duplicate junctions. This list of splice junctions was then used to create a genomic index for STAR second pass. All the samples were realigned, for the second time, with the new index information in order to increase the mapping quantity. The properly paired and discordantly paired aligned reads were merged, and duplicate reads were removed. The reads with N cigars were then split into exon fragments using splitNreads and sorted according to the read co-ordinates.^14^ INDELs were then left-aligned using Freebayes (v0.9.20) followed by variant calling.^4,5,15^ Preliminary variant calling was done with 4x read depth, base quality of 13 and minor allele frequency >1% and was followed by functional annotation.^6^

## Criteria for Variant Filtering

Several filtering criteria were applied in order to find the optimum balance between eliminating false positives variants and retaining true positives (Figure 2). In our RNA-Seq pipeline, we lowered the threshold of the variant callers’ filtering parameters to avoid losing putative variants. The variants with a read depth of less than 10x were then filtered out. Variants annotated in the RADAR database were removed, as they contain known RNA editing sites.^16^ Additionally, Regions defined by UCSC as simple tandem repeats were excluded.^17^ All the detected variants were called again using SAMtools (v0.1.19) and BCFtools (v0.1.19) for the purpose of determining mapping quality, base quality, strand and tail distance biases and were filtered accordingly.^18^ Variants in regions having more than 1/3^rd^ low mapping quality reads were also removed (Supplementary Fig. S5). Furthermore, a window spanning from 25 bases upstream to 25 bases downstream of the variants of interest was scanned for alternate allele bases. The proportion of alternate allele bases to reference allele bases was calculated and variants in regions with more than 1% alternate bases (error-prone regions) were filtered out as well. In addition, the absolute number of bases was counted for the regions 10 bases upstream and downstream of the variants. Variants where the upstream or downstream region contained less than 50% of the opposite region were excluded. Both primary and validation cohorts were processed in similar manner except for recalling variants using SAMtools and BCFtools. The VAF differences between DNA and RNA for the 9 recurrently mutated genes with significant WAI are shown in Supplementary Fig. S8.

# Supplementary Figures


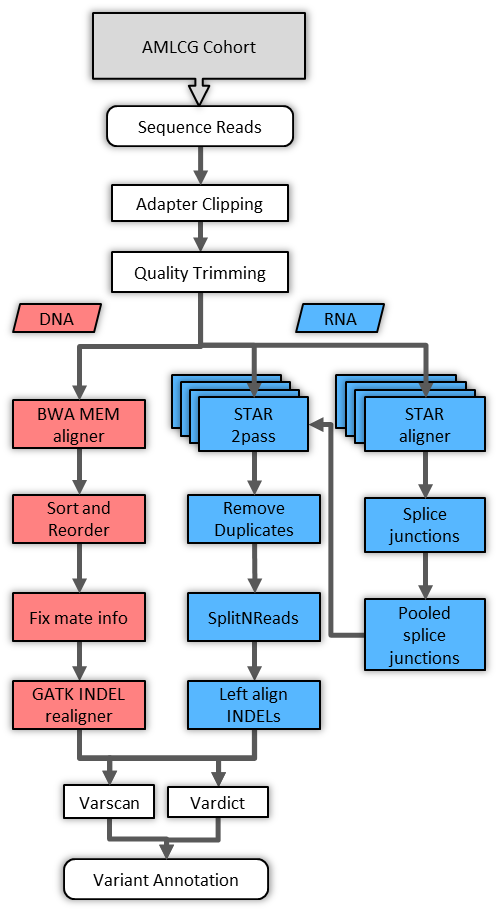


**Supplementary Figure S1:** Sequencing processing and variant calling pipeline.


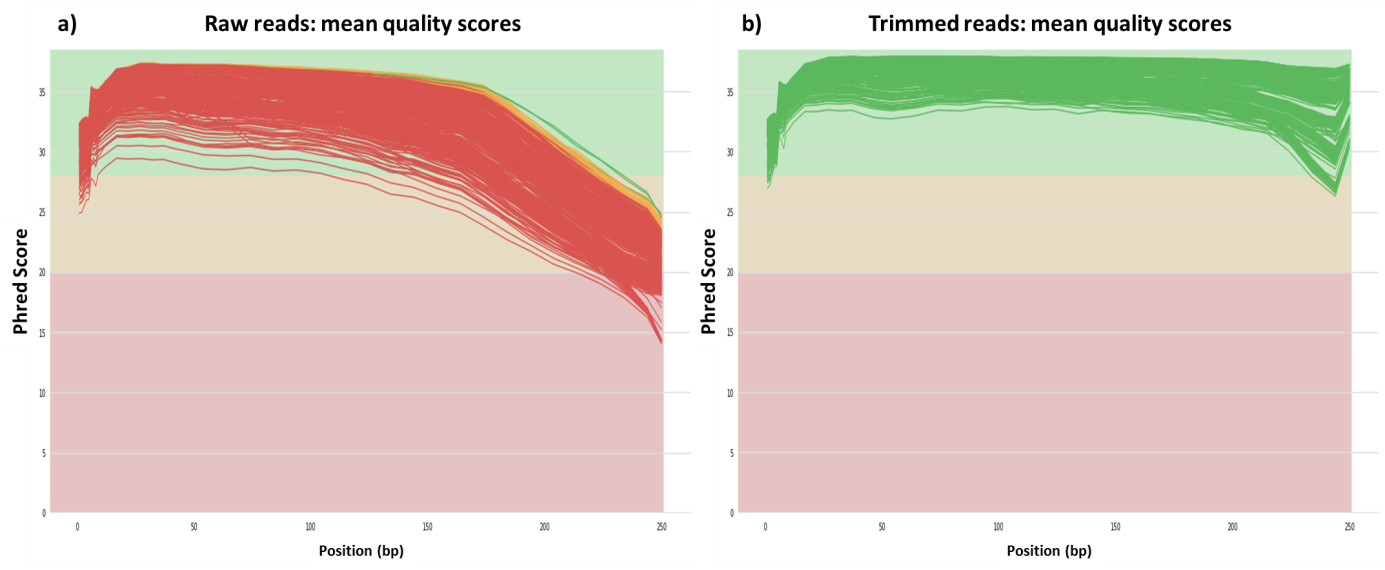


**Supplementary Figure S2:** DNA-Seq quality information of (a) raw and (b) quality trimmed reads per base level in AMLCG (n=246). The green, yellow and red colors indicate the high, medium and low-quality reads.

**
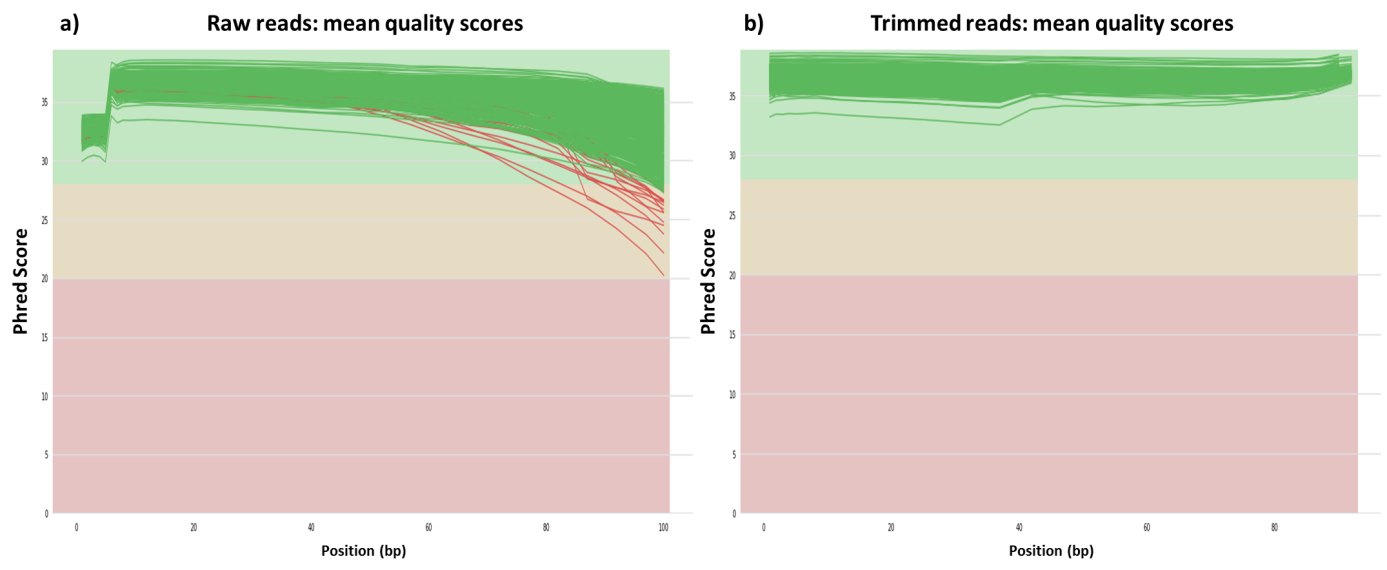
**

**Supplementary Figure S3:** RNA-Seq quality information of (a) raw and (b) quality trimmed reads per base level in the AMLCG cohort (n=246). The green, yellow and red colors indicate the high, medium and low-quality reads.

**Supplementary Figure S4:** RNA-Seq read depth of different variant classes for SNVs (a,c) and for INDELs (b,c). Note that no SNVs were detected at the read depth of 4x in the RNA seq, therefore the x axis of (a,c) starts at 5x.


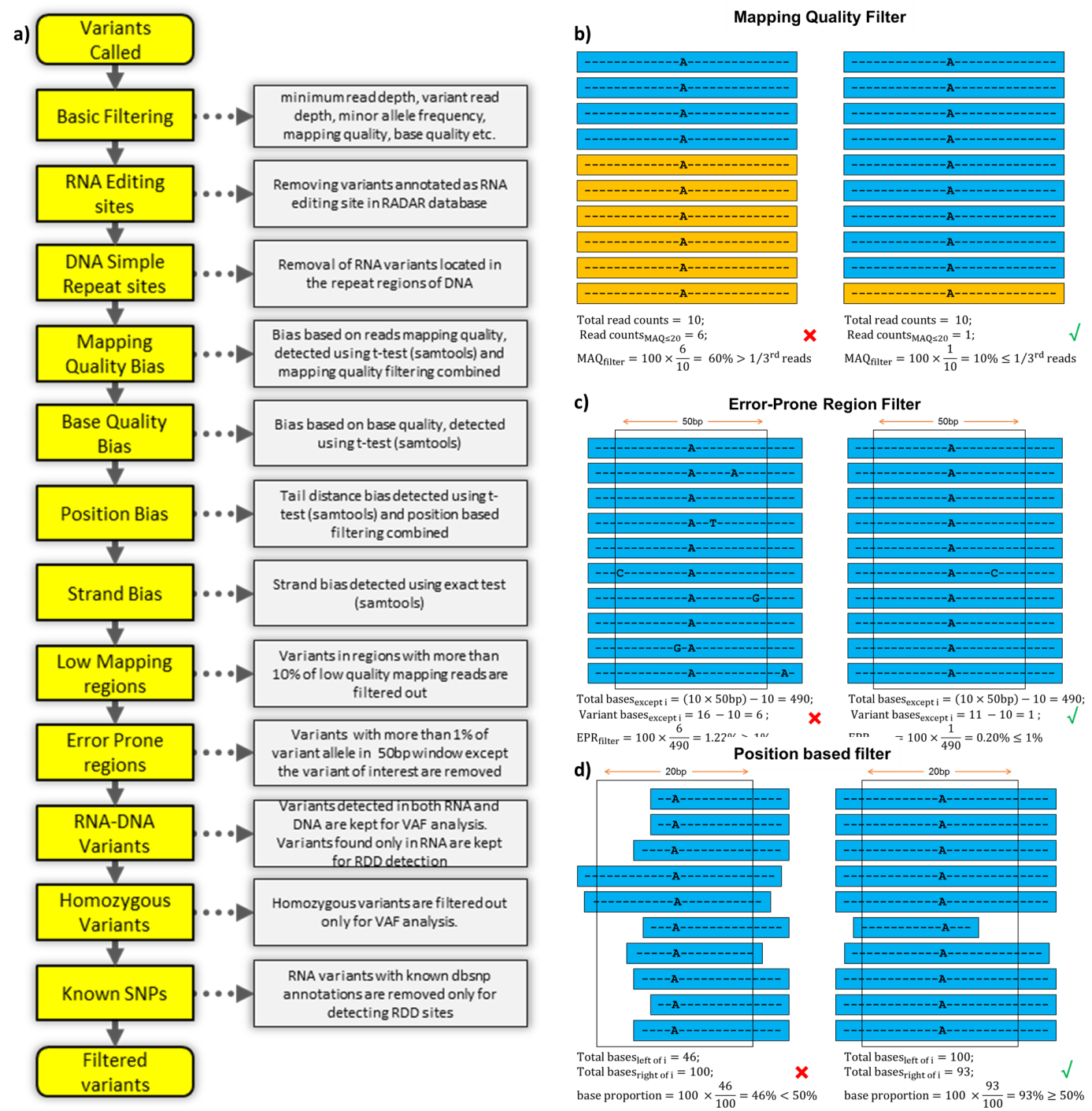


**Supplementary Figure S5:** Filtering criteria and visualization of criteria definitions. A) List of criteria applied. B) Low mapping quality region cut-off >=1/3rd reads. C) Error-prone region cut-off <=1% alternate allele frequency. D) Position based filtering cut-off >=50%.

**Supplementary Figure S6:** Filtering criteria applied on called variants. (a) Filtering categories: SB – Strand Bias; BQB – Base Quality Bias; MQB – Mapping Quality Bias; PB – Position Bias; MRD – Minimum Read Depth; EPR – Error-prone Region; EL/RL – Edit Loci/Repeat Loci. (b) Summary of filtered variants for SNVs and INDELs separately. Variant filter status was defined as ‘Unknown’ when the variants were unable to be subjected to defined filtering criteria mostly due to the absence of either forward or reverse stranded reads supporting each allele.

**Supplementary Figure S7:** Variant allele frequency differences between VarScan and VarDict for DNA (a) and RNA (b). The diagonal lines represent the expected variant VAF trend. The discrepancies in the INDEL VAFs led to the selection of VarDict as variant caller for INDELs. In the case of SNVs, the detected variants had very small mean difference between VarScan and VarDict VAFs of in the case of both DNA-Seq (µ = - 0.28, sd = 1.28) and RNA-Seq (µ = 0.18, sd = 1.05).

**Supplementary Figure S8:** Variant allele frequency differences between DNA and RNA for the 9 recurrently mutated genes with significant WAI in AMLCG cohort. The grey points represent the distribution of recurrent mutations in all the selected genes. Preferential expression of the mutant allele is observed in most genes. In the case of *WT1*, although most of the mutations are on the diagonal line, the model showed imbalance towards wild-type allele. This is due to the effect of difference in the coverage between the DNA- and RNA-Seq. The transformation of VAFs into expected and observed RNA variant read depth before applying the regression model statistically enriched the detection of this minor effect size. This influence can also be observed in the case of *CEBPA*.

# Supplementary Table

**Supplementary Table S1:** DNA and RNA sequence information in AMLCG.

| **Info** | **Targeted DNA-Seq** | **Total RNA-Seq** |
| --- | --- | --- |
| **Sequencing kit** | custom amplicon-based targeted enrichment assay (Haloplex) | Lexogen SENSE mRNA-Seq kit V2 |
| **Sequence length** | 250bp | 100bp |
| **Reference genome build** | hg19 (Human) | hg19 (Human) |
| **Average Total Aligned Reads (range)** | 722755 (301046–2208674) | 57568431 (36418879–167296173) |
| **Properly Paired Reads** | 99.6 % | 99.4 % |
| **Average Coverage in Target Regions (range)** | 542.3x (196.3 x – 2653.5 x) | 85.32x (33.2x – 301.4x) |
| **Average of Mean Insert Size (range)** | 178.6 (166.6 – 193.2) | 308.0 (134.9 – 583.9) |

**Supplementary Table S2:** List of genes and the regions of interest analysed using targeted DNA-Seq.^19^ CDS represent the coding sequence of the gene.

| **Gene** | **Region** | **Gene** | **Region** | **Gene** | **Region** |
| --- | --- | --- | --- | --- | --- |
| *ABCB1* | entire CDS | *GATA1* | exons 2,3 | *PTEN* | entire CDS |
| *ABCG2* | entire CDS | *GATA2* | exons 4-6 | *PTPN11* | exons 3,13 |
| *ADA* | entire CDS | *GATA3* | exons 3-5 | *PTPRT* | entire CDS |
| *ASXL1* | exon 12 | *HNRNPK* | entire CDS | *RAD21* | entire CDS |
| *BCOR* | entire CDS | *HRAS* | exons 2,3 | *RUNX1* | entire CDS |
| *BCORL1* | entire CDS | *IDH1* | exon 4 | *SETBP1* | exon 4 |
| *BRAF* | exons 11,12,15 | *IDH2* | exon 4 | *SF1* | entire CDS |
| *CBL* | exons 8,9 | *IL7R* | exon 6 | *SF3A1* | entire CDS |
| *CDA* | entire CDS | *JAK1* | exons 13-15 | *SF3B1* | exons 14-16 |
| *CDKN2A* | entire CDS | *JAK2* | exons 12-16 | *SMC1A* | entire CDS |
| *CEBPA* | entire CDS | *JAK3* | entire CDS | *SMC3* | entire CDS |
| *CSF3R* | exons 12-16 | *KDM6A* | entire CDS | *SRSF2* | exon 1 |
| *CSFR1* | exons 7, 22 | *KIT* | exons 8,9,11,17 | *STAG2* | entire CDS |
| *DAXX* | entire CDS | *KMT2A* | exons 1,3,4,33 | *TERC* | entire CDS |
| *DCK* | entire CDS | *KRAS* | exons 2,3 | *TERT* | exons 1,15 |
| *DCLK1* | entire CDS | *MIR-142* | entire CDS | *TET2* | entire CDS |
| *DIS3* | entire CDS | *MPL* | exon 10 | *TP53* | entire CDS |
| *DNMT3A* | exons 7-23 | *MYD88* | exons 3-5 | *U2AF1* | exons 2,6 |
| *ETV6* | entire CDS | *NOTCH1* | exons 26-28,34 | *U2AF2* | entire CDS |
| *EZH2* | entire CDS | *NPM1* | exons 10,11 | *WAC* | entire CDS |
| *FAM5C* | entire CDS | *NRAS* | exons 2,3 | *WT1* | entire CDS |
| *FBXW7* | exons 8-12 | *NT5C2* | entire CDS | *ZRSR2* | entire CDS |
| *FLT3* | exons 13-16, 20 | *PHF6* | entire CDS |  |  |

**Supplementary Table S3:** Common SNPs in the AMLCG cohort without recurrent mutations in the respective genes.

| Gene | Rsid | Chr | Pos | Ref | Alt | Type | Location | DNA VAF | Observed RNA variant read depth | Observed RNA total read depth | Expected RNA variant read depth |
| --- | --- | --- | --- | --- | --- | --- | --- | --- | --- | --- | --- |
| GATA2 | rs34172218 | chr3 | 128200072 | C | T | synonymous SNV | exonic | 46.79 | 15 | 23 | 10.76 |
| GATA2 | rs34172218 | chr3 | 128200072 | C | T | synonymous SNV | exonic | 48.74 | 22 | 40 | 19.5 |
| GATA2 | rs34172218 | chr3 | 128200072 | C | T | synonymous SNV | exonic | 46 | 11 | 22 | 10.12 |
| GATA2 | rs34172218 | chr3 | 128200072 | C | T | synonymous SNV | exonic | 44.56 | 18 | 18 | 8.02 |
| GATA2 | rs34172218 | chr3 | 128200072 | C | T | synonymous SNV | exonic | 52.98 | 0 | 13 | 6.89 |
| TET2 | rs12498609 | chr4 | 106155185 | C | G | nonsynonymous SNV | exonic | 46.72 | 6 | 11 | 5.14 |
| TET2 | rs111948941 | chr4 | 106155199 | C | T | nonsynonymous SNV | exonic | 51.95 | 6 | 10 | 5.2 |
| TET2 | rs111948941 | chr4 | 106155199 | C | T | nonsynonymous SNV | exonic | 48.15 | 6 | 11 | 5.3 |
| TET2 | rs111948941 | chr4 | 106155199 | C | T | nonsynonymous SNV | exonic | 47.48 | 16 | 37 | 17.57 |
| TET2 | rs6843141 | chr4 | 106155751 | G | A | nonsynonymous SNV | exonic | 44.86 | 7 | 13 | 5.83 |
| TET2 | rs61744960 | chr4 | 106156163 | G | A | nonsynonymous SNV | exonic | 46.93 | 6 | 12 | 5.63 |
| TET2 | rs61744960 | chr4 | 106156163 | G | A | nonsynonymous SNV | exonic | 49.52 | 8 | 13 | 6.44 |
| TET2 | rs61744960 | chr4 | 106156163 | G | A | nonsynonymous SNV | exonic | 49.84 | 8 | 16 | 7.97 |
| TET2 | rs61744960 | chr4 | 106156163 | G | A | nonsynonymous SNV | exonic | 44.88 | 9 | 14 | 6.28 |
| TET2 | rs61744960 | chr4 | 106156163 | G | A | nonsynonymous SNV | exonic | 49.25 | 6 | 12 | 5.91 |
| TET2 | rs61744960 | chr4 | 106156163 | G | A | nonsynonymous SNV | exonic | 47.71 | 11 | 21 | 10.02 |
| TET2 | rs17253672 | chr4 | 106156187 | C | T | nonsynonymous SNV | exonic | 50.96 | 7 | 17 | 8.66 |
| TET2 | rs17253672 | chr4 | 106156187 | C | T | nonsynonymous SNV | exonic | 50.82 | 14 | 26 | 13.21 |
| TET2 | rs17253672 | chr4 | 106156187 | C | T | nonsynonymous SNV | exonic | 54.46 | 16 | 28 | 15.25 |
| TET2 | rs17253672 | chr4 | 106156187 | C | T | nonsynonymous SNV | exonic | 53.2 | 12 | 24 | 12.77 |
| TET2 | rs17253672 | chr4 | 106156187 | C | T | nonsynonymous SNV | exonic | 51.7 | 12 | 16 | 8.27 |
| TET2 | rs17253672 | chr4 | 106156187 | C | T | nonsynonymous SNV | exonic | 49.78 | 10 | 23 | 11.45 |
| TET2 | rs17253672 | chr4 | 106156187 | C | T | nonsynonymous SNV | exonic | 48.45 | 10 | 17 | 8.24 |
| TET2 | rs17253672 | chr4 | 106156187 | C | T | nonsynonymous SNV | exonic | 47.42 | 15 | 29 | 13.75 |
| TET2 | rs17253672 | chr4 | 106156187 | C | T | nonsynonymous SNV | exonic | 48.76 | 6 | 11 | 5.36 |
| TET2 | rs17253672 | chr4 | 106156187 | C | T | nonsynonymous SNV | exonic | 52.85 | 12 | 19 | 10.04 |
| TET2 | rs17253672 | chr4 | 106156187 | C | T | nonsynonymous SNV | exonic | 51.28 | 12 | 21 | 10.77 |
| TET2 | rs17253672 | chr4 | 106156187 | C | T | nonsynonymous SNV | exonic | 54.07 | 7 | 11 | 5.95 |
| TET2 | rs17253672 | chr4 | 106156187 | C | T | nonsynonymous SNV | exonic | 42.87 | 20 | 46 | 19.72 |
| TET2 | rs17253672 | chr4 | 106156187 | C | T | nonsynonymous SNV | exonic | 45.5 | 8 | 13 | 5.92 |
| TET2 | rs17253672 | chr4 | 106156187 | C | T | nonsynonymous SNV | exonic | 46.03 | 17 | 28 | 12.89 |
| TET2 | rs17253672 | chr4 | 106156187 | C | T | nonsynonymous SNV | exonic | 51.87 | 20 | 30 | 15.56 |
| TET2 | rs17253672 | chr4 | 106156187 | C | T | nonsynonymous SNV | exonic | 48.28 | 9 | 15 | 7.24 |
| TET2 | rs144386291 | chr4 | 106157698 | T | C | nonsynonymous SNV | exonic | 52.82 | 8 | 13 | 6.87 |
| TET2 | rs144386291 | chr4 | 106157698 | T | C | nonsynonymous SNV | exonic | 42.34 | 10 | 19 | 8.04 |
| TET2 | rs3796927 | chr4 | 106158216 | G | A | synonymous SNV | exonic | 54.29 | 8 | 12 | 6.51 |
| TET2 | rs3796927 | chr4 | 106158216 | G | A | synonymous SNV | exonic | 49.69 | 8 | 17 | 8.45 |
| TET2 | rs75056899 | chr4 | 106158350 | A | C | nonsynonymous SNV | exonic | 50.79 | 20 | 33 | 16.76 |
| TET2 | rs75056899 | chr4 | 106158350 | A | C | nonsynonymous SNV | exonic | 50.29 | 13 | 26 | 13.08 |
| TET2 | rs75056899 | chr4 | 106158350 | A | C | nonsynonymous SNV | exonic | 55.51 | 8 | 26 | 14.43 |
| TET2 | rs62623390 | chr4 | 106196770 | G | A | nonsynonymous SNV | exonic | 44.37 | 13 | 25 | 11.09 |
| TET2 | rs62623390 | chr4 | 106196770 | G | A | nonsynonymous SNV | exonic | 47.69 | 10 | 19 | 9.06 |
| TET2 | rs142312318 | chr4 | 106196819 | G | T | nonsynonymous SNV | exonic | 50.53 | 14 | 22 | 11.12 |
| TET2 | rs142312318 | chr4 | 106196819 | G | T | nonsynonymous SNV | exonic | 46.2 | 9 | 17 | 7.85 |
| TET2 | rs34402524 | chr4 | 106196829 | T | G | nonsynonymous SNV | exonic | 49.32 | 9 | 22 | 10.85 |
| TET2 | rs34402524 | chr4 | 106196829 | T | G | nonsynonymous SNV | exonic | 50.44 | 12 | 25 | 12.61 |
| TET2 | rs34402524 | chr4 | 106196829 | T | G | nonsynonymous SNV | exonic | 48.8 | 12 | 21 | 10.25 |
| TET2 | rs34402524 | chr4 | 106196829 | T | G | nonsynonymous SNV | exonic | 44.46 | 11 | 16 | 7.11 |
| TET2 | rs34402524 | chr4 | 106196829 | T | G | nonsynonymous SNV | exonic | 57.21 | 12 | 22 | 12.59 |
| TET2 | rs34402524 | chr4 | 106196829 | T | G | nonsynonymous SNV | exonic | 52.44 | 7 | 16 | 8.39 |
| TET2 | rs34402524 | chr4 | 106196829 | T | G | nonsynonymous SNV | exonic | 50 | 16 | 34 | 17 |
| TET2 | rs34402524 | chr4 | 106196829 | T | G | nonsynonymous SNV | exonic | 50.29 | 9 | 19 | 9.56 |
| TET2 | rs34402524 | chr4 | 106196829 | T | G | nonsynonymous SNV | exonic | 52.25 | 7 | 16 | 8.36 |
| TET2 | rs34402524 | chr4 | 106196829 | T | G | nonsynonymous SNV | exonic | 52.24 | 6 | 10 | 5.22 |
| TET2 | rs34402524 | chr4 | 106196829 | T | G | nonsynonymous SNV | exonic | 48.19 | 29 | 50 | 24.09 |
| TET2 | rs34402524 | chr4 | 106196829 | T | G | nonsynonymous SNV | exonic | 50.65 | 9 | 16 | 8.1 |
| TET2 | rs34402524 | chr4 | 106196829 | T | G | nonsynonymous SNV | exonic | 51.6 | 32 | 58 | 29.93 |
| TET2 | rs34402524 | chr4 | 106196829 | T | G | nonsynonymous SNV | exonic | 51 | 6 | 13 | 6.63 |
| TET2 | rs34402524 | chr4 | 106196829 | T | G | nonsynonymous SNV | exonic | 55.25 | 6 | 15 | 8.29 |
| TET2 | rs34402524 | chr4 | 106196829 | T | G | nonsynonymous SNV | exonic | 56.85 | 16 | 26 | 14.78 |
| TET2 | rs34402524 | chr4 | 106196829 | T | G | nonsynonymous SNV | exonic | 48.26 | 7 | 12 | 5.79 |
| TET2 | rs34402524 | chr4 | 106196829 | T | G | nonsynonymous SNV | exonic | 52.57 | 11 | 23 | 12.09 |
| TET2 | rs34402524 | chr4 | 106196829 | T | G | nonsynonymous SNV | exonic | 47.49 | 6 | 13 | 6.17 |
| TET2 | rs34402524 | chr4 | 106196829 | T | G | nonsynonymous SNV | exonic | 50.26 | 8 | 19 | 9.55 |
| TET2 | rs146348065 | chr4 | 106196834 | C | T | nonsynonymous SNV | exonic | 42.93 | 8 | 27 | 11.59 |
| TET2 | rs2454206 | chr4 | 106196951 | A | G | nonsynonymous SNV | exonic | 52.3 | 14 | 32 | 16.74 |
| TET2 | rs2454206 | chr4 | 106196951 | A | G | nonsynonymous SNV | exonic | 53.81 | 9 | 12 | 6.46 |
| TET2 | rs2454206 | chr4 | 106196951 | A | G | nonsynonymous SNV | exonic | 45.28 | 6 | 12 | 5.43 |
| TET2 | rs2454206 | chr4 | 106196951 | A | G | nonsynonymous SNV | exonic | 48.11 | 19 | 30 | 14.43 |
| TET2 | rs2454206 | chr4 | 106196951 | A | G | nonsynonymous SNV | exonic | 53.48 | 8 | 12 | 6.42 |
| TET2 | rs2454206 | chr4 | 106196951 | A | G | nonsynonymous SNV | exonic | 50.87 | 12 | 23 | 11.7 |
| TET2 | rs2454206 | chr4 | 106196951 | A | G | nonsynonymous SNV | exonic | 50.89 | 13 | 27 | 13.74 |
| TET2 | rs2454206 | chr4 | 106196951 | A | G | nonsynonymous SNV | exonic | 49.64 | 6 | 10 | 4.96 |
| TET2 | rs2454206 | chr4 | 106196951 | A | G | nonsynonymous SNV | exonic | 48.85 | 9 | 15 | 7.33 |
| TET2 | rs2454206 | chr4 | 106196951 | A | G | nonsynonymous SNV | exonic | 53.11 | 10 | 19 | 10.09 |
| TET2 | rs2454206 | chr4 | 106196951 | A | G | nonsynonymous SNV | exonic | 48.5 | 8 | 21 | 10.19 |
| TET2 | rs2454206 | chr4 | 106196951 | A | G | nonsynonymous SNV | exonic | 49.37 | 13 | 23 | 11.36 |
| TET2 | rs2454206 | chr4 | 106196951 | A | G | nonsynonymous SNV | exonic | 48.66 | 11 | 23 | 11.19 |
| TET2 | rs2454206 | chr4 | 106196951 | A | G | nonsynonymous SNV | exonic | 49.15 | 6 | 13 | 6.39 |
| TET2 | rs2454206 | chr4 | 106196951 | A | G | nonsynonymous SNV | exonic | 46.93 | 7 | 13 | 6.1 |
| TET2 | rs2454206 | chr4 | 106196951 | A | G | nonsynonymous SNV | exonic | 43.07 | 11 | 21 | 9.04 |
| TET2 | rs2454206 | chr4 | 106196951 | A | G | nonsynonymous SNV | exonic | 48.3 | 9 | 18 | 8.69 |
| TET2 | rs2454206 | chr4 | 106196951 | A | G | nonsynonymous SNV | exonic | 48.02 | 13 | 40 | 19.21 |
| TET2 | rs2454206 | chr4 | 106196951 | A | G | nonsynonymous SNV | exonic | 44.61 | 11 | 17 | 7.58 |
| TET2 | rs2454206 | chr4 | 106196951 | A | G | nonsynonymous SNV | exonic | 46.14 | 7 | 19 | 8.77 |
| TET2 | rs2454206 | chr4 | 106196951 | A | G | nonsynonymous SNV | exonic | 46.81 | 7 | 18 | 8.43 |
| TET2 | rs2454206 | chr4 | 106196951 | A | G | nonsynonymous SNV | exonic | 49.6 | 20 | 33 | 16.37 |
| TET2 | rs2454206 | chr4 | 106196951 | A | G | nonsynonymous SNV | exonic | 47.63 | 23 | 34 | 16.19 |
| TET2 | rs2454206 | chr4 | 106196951 | A | G | nonsynonymous SNV | exonic | 47.43 | 9 | 13 | 6.17 |
| TET2 | rs2454206 | chr4 | 106196951 | A | G | nonsynonymous SNV | exonic | 51.77 | 8 | 15 | 7.77 |
| TET2 | rs2454206 | chr4 | 106196951 | A | G | nonsynonymous SNV | exonic | 50.23 | 6 | 15 | 7.53 |
| TET2 | rs2454206 | chr4 | 106196951 | A | G | nonsynonymous SNV | exonic | 49.03 | 6 | 14 | 6.86 |
| TET2 | rs62621450 | chr4 | 106197000 | A | G | nonsynonymous SNV | exonic | 46.52 | 7 | 16 | 7.44 |
| TET2 | rs62621450 | chr4 | 106197000 | A | G | nonsynonymous SNV | exonic | 51.48 | 10 | 19 | 9.78 |
| WT1 | rs2234583 | chr11 | 32456298 | G | A | synonymous SNV | exonic | 43.8 | 12 | 14 | 6.13 |
| WT1 | rs2234583 | chr11 | 32456298 | G | A | synonymous SNV | exonic | 47.02 | 20 | 42 | 19.75 |
| WT1 | rs2234583 | chr11 | 32456298 | G | A | synonymous SNV | exonic | 53.26 | 11 | 22 | 11.72 |
| WT1 | rs2234583 | chr11 | 32456298 | G | A | synonymous SNV | exonic | 48.76 | 27 | 42 | 20.48 |
| WT1 | rs2234583 | chr11 | 32456298 | G | A | synonymous SNV | exonic | 47.51 | 6 | 14 | 6.65 |
| SRSF2 | rs237057 | chr17 | 74733099 | G | A | synonymous SNV | exonic | 48.26 | 32 | 56 | 27.03 |
| SRSF2 | rs237057 | chr17 | 74733099 | G | A | synonymous SNV | exonic | 48.01 | 11 | 18 | 8.64 |
| SRSF2 | rs237057 | chr17 | 74733099 | G | A | synonymous SNV | exonic | 48.63 | 44 | 80 | 38.9 |
| SRSF2 | rs237057 | chr17 | 74733099 | G | A | synonymous SNV | exonic | 51.99 | 53 | 109 | 56.67 |
| SRSF2 | rs237057 | chr17 | 74733099 | G | A | synonymous SNV | exonic | 50.62 | 38 | 64 | 32.4 |
| SRSF2 | rs237057 | chr17 | 74733099 | G | A | synonymous SNV | exonic | 50 | 33 | 78 | 39 |
| SRSF2 | rs237057 | chr17 | 74733099 | G | A | synonymous SNV | exonic | 44.09 | 10 | 30 | 13.23 |
| SRSF2 | rs237057 | chr17 | 74733099 | G | A | synonymous SNV | exonic | 47.74 | 6 | 15 | 7.16 |
| SRSF2 | rs237057 | chr17 | 74733099 | G | A | synonymous SNV | exonic | 47.62 | 10 | 16 | 7.62 |
| SRSF2 | rs237057 | chr17 | 74733099 | G | A | synonymous SNV | exonic | 52.4 | 10 | 24 | 12.58 |
| SRSF2 | rs237057 | chr17 | 74733099 | G | A | synonymous SNV | exonic | 51.35 | 23 | 60 | 30.81 |
| SRSF2 | rs237057 | chr17 | 74733099 | G | A | synonymous SNV | exonic | 48.79 | 26 | 58 | 28.3 |
| SRSF2 | rs237057 | chr17 | 74733099 | G | A | synonymous SNV | exonic | 51.89 | 17 | 40 | 20.76 |
| SRSF2 | rs237057 | chr17 | 74733099 | G | A | synonymous SNV | exonic | 51.64 | 12 | 21 | 10.84 |
| SRSF2 | rs237057 | chr17 | 74733099 | G | A | synonymous SNV | exonic | 50 | 19 | 51 | 25.5 |
| SRSF2 | rs237057 | chr17 | 74733099 | G | A | synonymous SNV | exonic | 49.4 | 38 | 80 | 39.52 |
| SRSF2 | rs237057 | chr17 | 74733099 | G | A | synonymous SNV | exonic | 51.46 | 22 | 36 | 18.53 |
| SRSF2 | rs237057 | chr17 | 74733099 | G | A | synonymous SNV | exonic | 51.39 | 8 | 19 | 9.76 |
| SRSF2 | rs237057 | chr17 | 74733099 | G | A | synonymous SNV | exonic | 45.16 | 17 | 30 | 13.55 |
| SRSF2 | rs237057 | chr17 | 74733099 | G | A | synonymous SNV | exonic | 48.48 | 35 | 72 | 34.91 |
| RUNX1 | rs111527738 | chr21 | 36259324 | A | G | nonsynonymous SNV | exonic | 49.51 | 6 | 17 | 8.42 |
| RUNX1 | rs111527738 | chr21 | 36259324 | A | G | nonsynonymous SNV | exonic | 50.14 | 47 | 77 | 38.61 |
| RUNX1 | rs111527738 | chr21 | 36259324 | A | G | nonsynonymous SNV | exonic | 44.75 | 41 | 93 | 41.62 |
| RUNX1 | rs111527738 | chr21 | 36259324 | A | G | nonsynonymous SNV | exonic | 49.66 | 54 | 164 | 81.44 |
| RUNX1 | rs111527738 | chr21 | 36259324 | A | G | nonsynonymous SNV | exonic | 47.38 | 38 | 77 | 36.48 |
| RUNX1 | rs111527738 | chr21 | 36259324 | A | G | nonsynonymous SNV | exonic | 50.42 | 28 | 52 | 26.22 |

# References

1. Bolger, A. M., Lohse, M. & Usadel, B. Trimmomatic: A flexible trimmer for Illumina sequence data. *Bioinformatics* **30,** 2114–2120 (2014).

2. Li, H. Aligning sequence reads, clone sequences and assembly contigs with BWA-MEM. (2013). doi:arXiv:1303.3997 [q-bio.GN]

3. McKenna, A. *et al.* The genome analysis toolkit: A MapReduce framework for analyzing next-generation DNA sequencing data. *Genome Res.* **20,** 1297–1303 (2010).

4. Koboldt, D. C., Larson, D. E. & Wilson, R. K. Using varscan 2 for germline variant calling and somatic mutation detection. *Curr. Protoc. Bioinforma.* (2013). doi:10.1002/0471250953.bi1504s44

5. Lai, Z. *et al.* VarDict: a novel and versatile variant caller for next-generation sequencing in cancer research. *Nucleic Acids Res.* **44,** e108 (2016).

6. Wang, K., Li, M. & Hakonarson, H. ANNOVAR: Functional annotation of genetic variants from high-throughput sequencing data. *Nucleic Acids Res.* **38,** e164 (2010).

7. Bamford, S. *et al.* The COSMIC (Catalogue of Somatic Mutations in Cancer) database and website. *Br. J. Cancer* **91,** 355–8 (2004).

8. Sherry, S. T. dbSNP: the NCBI database of genetic variation. *Nucleic Acids Res.* **29,** 308–311 (2001).

9. Landrum, M. J. *et al.* ClinVar: Public archive of relationships among sequence variation and human phenotype. *Nucleic Acids Res.* **42,** D980–D985 (2014).

10. Auton, A. *et al.* A global reference for human genetic variation. *Nature* **526,** 68–74 (2015).

11. Barreto, H. & Howland, F. Lexogen. *Introd. Econom.* **001,** 1–9

12. Martin, M. Cutadapt removes adapter sequences from high-throughput sequencing reads. *EMBnet.journal* **17,** 10 (2011).

13. Dobin, A. *et al.* STAR: Ultrafast universal RNA-seq aligner. *Bioinformatics* **29,** 15–21 (2013).

14. Ahdesmaki, M. SplitNRead. Available at: https://github.com/mjafin/splitNreads.

15. Garrison, E. & Marth, G. Haplotype-based variant detection from short-read sequencing. (2012). doi:arXiv:1207.3907 [q-bio.GN]

16. Ramaswami, G. & Li, J. B. RADAR: A rigorously annotated database of A-to-I RNA editing. *Nucleic Acids Res.* **42,** (2014).

17. Karolchik, D. The UCSC Table Browser data retrieval tool. *Nucleic Acids Res.* **32,** 493D–496 (2004).

18. Li, H. *et al.* The Sequence Alignment/Map format and SAMtools. *Bioinformatics* **25,** 2078–2079 (2009).

19. Metzeler, K. H. *et al.* Spectrum and prognostic relevance of driver gene mutations in acute myeloid leukemia. doi:10.1182/blood-2016-01
